# The Effects of Levosimendan on Microcirculation and Peripheral Perfusion in Septic Shock: A Pilot Study

**DOI:** 10.3390/life15060871

**Published:** 2025-05-28

**Authors:** Veronica Gagliardi, Francesco Ceccherelli, Antonello Lovato, Giuseppe Gagliardi

**Affiliations:** 1 Department of Anesthesiology and Intensive Care, University of Padova, 35122 Padova, Italy; giuseppe.gagliardi@aulss5.unipd.it; 2 AIRAS, 36045 Lonigo, Italy; fceccherelli@airas.it (F.C.); lovatoantonello@gmail.com (A.L.); 3 Department of Anesthesiology and Intensive Care, Ospedale Santa Maria della Misericordia, 45100 Rovigo, Italy

**Keywords:** microcirculation, inodilators, levosimendan, septic shock

## Abstract

Septic patients can show multiorgan failure even after an apparent recovery of hemodynamic stability. The underlying mechanism is unclear, but the main pathological element is microcirculation impairment, leading to insufficient oxygen delivery. This study aimed to assess the effects of levosimendan administration on peripheral perfusion in the prodromic phases of sepsis and compare them with the variations in microcirculation perfusion occurring with conventional dobutamine therapy. Sixteen patients with sepsis were enrolled, eight of whom were treated with norepinephrine and levosimendan and the other eight with norepinephrine and dobutamine. We observed a trend of reduction in the hematic lactate concentration and an increase in peripheral perfusion in the patients treated with levosimendan. The latter also occurred in the dobutamine group, although to a lower degree. Hematic lactate was significantly reduced in the levosimendan group, probably because of the enhanced aerobic metabolism, due to both the action on mitochondrial K_ATP_ channels and the better oxygen delivery to cells. The lactate values varied from T_0_ (2.28 ± 0.25 mmol/L) to T_2_ (1.45 ± 0.31 mmol/L) in the levosimendan group vs. from T_0_ (2.79 ± 0.91 mmol/L) to T_2_ (2.92 ± 0.76 mmol/) L in the dobutamine group. Hence, levosimendan may be indicated in septic patients with impaired microcirculation and tissue oxygenation and, consequently, high lactate levels. Further studies are needed to draw a profile of levosimendan as a possible treatment to restore microcirculation in septic patients.

## 1. Introduction

Sepsis is one of the most critical matters in current medicine due to the complexity of its pathophysiology and therapeutic approaches. It consists of life-threatening organ dysfunction caused by a dysregulated host response to infection. Septic shock is a subset of sepsis with circulatory and cellular/metabolic dysfunction associated with a higher risk of mortality.

Shock is defined as a condition in which there is an insufficient delivery of oxygen, where an imbalance between oxygen consumption and demand occurs. Septic shock is characterized by hypotension that is persistent after fluid resuscitation with signs of hypoperfusion and organ failure. Septic shock is distributive, and the main pathological mechanism is the formation of shunts [1,2,3].

Owing to missed diagnoses, epidemiologic studies deal with the “treated incidence” of sepsis cases, rather than the actual incidence. It is recorded in 2% of patients admitted to hospitals, half of which are treated in intensive care units, accounting for 10% of all ICU admissions [4,5,6,7,8]. Sepsis can also induce septic cardiomyopathy, with de novo acute heart failure, due to myocardial depression, contributing to a sepsis mortality rate of 30%. The prevalence of septic cardiomyopathy among septic patients widely varies (from 20% to 60%), and it is the main cause of the need for inotrope administration in septic shock [3].

The host response to sepsis consists of both pro-inflammatory and anti-inflammatory immunosuppressive reactions.

The direction, extent, and duration of these reactions are determined by both host factors (genetic factors, age, coexisting illnesses, and medications) and pathogen factors (microbial load and virulence). From a hemodynamic point of view, alterations lead to a significant decrease in vascular tone; a hypovolemic component resulting from relative or central hypovolemia; and fluid losses due to vascular leak, a variable degree of myocardial dysfunction, dysregulation of the regional blood flow distribution, and microvascular alterations. Perfusion abnormalities often persist despite achieving resuscitation targets, leading to the development of organ dysfunction [6,7,8,9,10]. The orthogonal polarization spectral imaging technique is employed to study microcirculation in humans to investigate the sublingual microcirculation, which is altered in patients with sepsis [11].

A vasomotion disequilibrium occurs in sepsis, characterized by non-specific systemic vasodilatation, pulmonary vasoconstriction, and increases in capillary permeability. This paralysis of the peripheral vascular system leads to impairment in the regulation and distribution of peripheral blood flow [7,8].

Moreover, sepsis is associated with a loss of endothelium structure, increasing vascular permeability. This dysregulation of endothelial cells is also associated with a procoagulant and pro-inflammatory state, with the secretion of adhesion molecules, contributing to the alterations in perfusion and an impaired sensitivity to vasodilating and vasoconstrictive substances. In addition, the glycocalyx is degraded; the severity of its breakdown is associated with poor clinical outcomes.

An intact and functional microcirculatory system is essential for adequate oxygen delivery in the peripheral tissues [9]. Therefore, an impaired microcirculation damages the organ perfusion despite the macrocirculation being preserved and the parameters staying within the normal range, as conventional hemodynamic resuscitation may not correct microcirculatory alterations [8,12].

Keeping an optimal peripheral perfusion minimizes hypoxia-induced cellular damage. A non-invasive assessment of muscular microcirculation can be performed with the laser Doppler technique.

In this context, inodilators could play a pivotal role in restoring microcirculation function, whereas vasopressor administration alone could detrimentally affect the oxygen consumption/delivery balance. Leading to both cardiac output augmentation and peripheral vasodilation, inodilatory agents can increase tissue oxygenation, which is fundamental for restoring organ function [13,14].

Among inodilatory agents, levosimendan leads to the sensitization of troponin C to calcium in cardiac muscle, exerting a positive inotropic effect without increasing myocardial oxygen consumption [15]. Furthermore, the drug opens adenosine triphosphate-sensitive potassium (K_ATP_) channels in vascular smooth muscle cells and induces the vasodilation of the pulmonary, coronary, and peripheral arteries and venous circulation. Levosimendan also opens mitochondrial K channels and exerts an organ-protective effect. At higher doses, it also acts as a phosphodiesterase type 3 (PDE3) inhibitor [16]. Levosimendan does not stimulate adrenergic receptors, causes little increase in myocardial oxygen consumption, and may, contrarily, have a cardioprotective effect. Furthermore, it improves diastolic function (a key element of septic cardiomyopathy) to a better extent than dobutamine and improves ventriculoarterial coupling. Levosimendan infusion has been demonstrated to improve microcirculation perfusion and restore the muscular lactate/pyruvate ratio, leading to aerobic metabolism. Binding to the K_ATP_ channels located in mitochondria, the drug can protect mitochondria from oxidative injury, preventing calcium overload and thereby reducing mitochondrial ROS generation [16]. Thanks to its vasodilatory and pleiotropic effects, levosimendan enhances both convection and diffusion (increased perfused vessel density) in microcirculation, reducing the oxygen diffusion distance and improving cellular oxygen delivery and availability [17,18,19]. Nevertheless, most studies, including the Levosimendan for the Prevention of Acute Organ Dysfunction in Sepsis (LeoPARDS) trial, have shown that adding levosimendan to standard management does not result in less severe organ dysfunction or mortality. Moreover, the ICU stay duration is not reduced, although levosimendan is related to a higher reduction in lactate levels and improvement in cardiac function. There is no evidence of the superiority of levosimendan over dobutamine in patients with sepsis and septic shock [20,21,22].

However, studies show heterogeneity in their mortality data, which hinders their interpretation. Moreover, the individual clinical trials are small and employ different treatment protocols. We can, therefore, affirm that levosimendan offers no advantage over dobutamine in septic shock patients in terms of mortality and the length of ICU stays, even though it improves the metabolic state and cardiac function [22].

### Aim of the Study

The aim of this study was to assess the effects of levosimendan administration on peripheral perfusion associated with the infusion of norepinephrine in the prodromic phases of sepsis. We compared them with the variations in microcirculation perfusion occurring with norepinephrine associated with dobutamine. Given levosimendan’s mechanisms of action, we believe it could restore peripheral perfusion and induce a greater vasodilatory effect than dobutamine.

## 2. Materials and Methods

We enrolled 16 patients admitted to intensive care units for severe sepsis who underwent the early treatment and fluid resuscitation indicated by the international guidelines of the Surviving Sepsis Campaign of 2021. We monitored the adequate volemia with the PiCCO system. The patients were euvolemic and maintained their sinus rhythm, not presenting arrhythmic phenomena. All enrolled patients had been intubated and mechanically ventilated. Informed consent for their inclusion in this study was obtained from all subjects involved. We excluded patients with pulmonary sepsis, with a previous diagnosis of peripheral arteriopathy, and affected by Raynaud’s phenomenon.

The patients were then divided into 2 groups.

Group A comprised 8 patients treated with levosimendan (with a bolus of 12 mcg/kg followed by a continuous infusion of 0.1 mcg/kg/min for 24 h) and a continuous infusion of noradrenaline, with a maximum dosage of 0.4 mcg/kg/min. The patients included in this group had an indication for levosimendan treatment because they showed a reduced cardiac output (Table 1).

Group B included 8 patients treated with 5 mcg/kg/min of dobutamine and noradrenaline at the same dosage as the previous group (Table 2).

The two groups were homogeneous concerning diagnosis, age, and sex. As a main indication of levosimendan is a depressed EF, the main difference between the two groups was the cardiological disease. Nevertheless, our primary endpoint was the assessment of microcirculation activity caused by the drugs’ administration to compare the two groups.

We employed either dobutamine or levosimendan, focusing on their dilatory function in microcirculation. Although all patients had systolic dysfunction, it was documented before the onset of the septic shock in the levosimendan group, whereas it was only induced by septic shock in the dobutamine group. Patients did not receive any other vasopressor or inotropic support besides the mentioned drugs.

**Figure 1:**
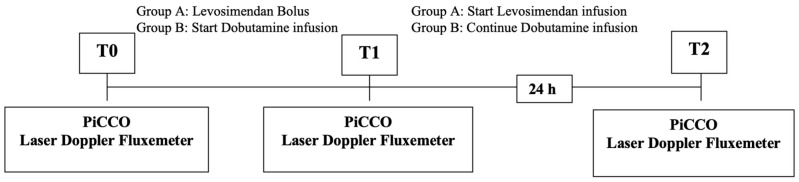
Timetable.

The outcomes of the two treatments were analyzed by detecting the variations in the hemodynamic parameters measured with the PiCCO system and assessing the peripheral blood flow with Periflux, a laser Doppler fluxmeter (Figure 1).

The hemodynamic parameters (PAM, CI, SvO_2_, lactate, and RVSI) were measured for each patient at the beginning, T_0_ (=basal), at T_1_ (=after the administration of the bolus of levosimendan in Group A patients and after starting the dobutamine infusion in Group B patients), and at T_2_ (=after 24 h) using the PiCCO monitor (pulse index continuous cardiac output) [23].

**Figure 2:**
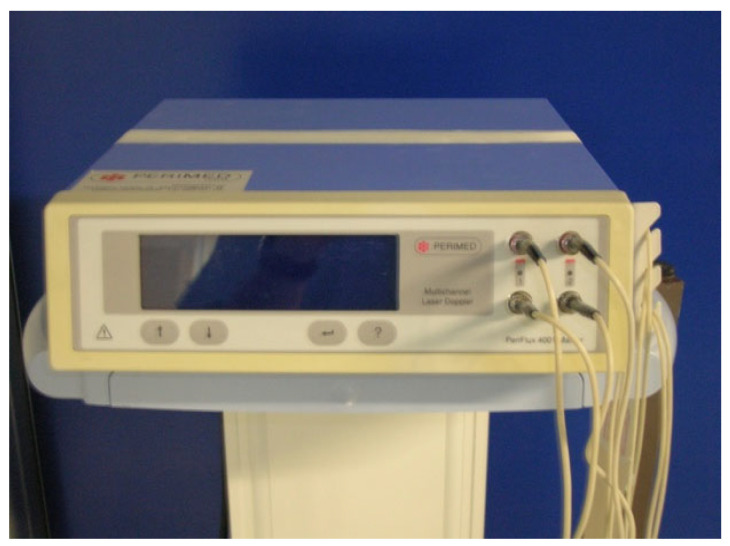
Laser Doppler fluxmeter.

The peripheral blood flow assessment was performed with the laser Doppler fluxmeter PERIFLUX PF 4001 MASTER^®^, manufactured by PERIMED, Järfälla, Sweden, a laser diode system producing non-collimated light at 780 nm, emitting a maximal radiation of 0.8 mW, with a range of measurement of Doppler variation from 20 Hz to 24 kHz. All data were collected employing the same kind of probe (PF 408), maintaining the same setting during the time (0.2 s), and keeping the same measurement scale. The environmental temperature was kept between 25 and 26 °C during all the measurement times. The measurements were performed in all patients at times T_0_, T_1_, and T_2_ on the upper limb (proximally on the deltoid muscle and distally on the extensor carpi radialis longus muscle) and the lower limb (proximally on the vastus lateralis and distally on the anterior tibialis muscle). The Periflux system allows the measurement of cutaneous microcirculation by using the Doppler effect. The probe emits a laser light beam toward the skin. The beam is reflected and divided into two components when it hits the body: the first consists of the laser light, which hits the static objects and is reflected with the same frequency as the incident ray; the second component is reflected after the interaction with the moving objects, in this case, red blood cells. Its frequency varies according to the Doppler effect, as analyzed with the probe. The product of multiplication between the velocity (v) and concentration of moving red blood cells (CMBCs) represents the flow, described as the perfusion unit (PU) [24,25] (Figure 2).

## 3. Results

The results are reported in Table 3, Table 4, Table 5 and Table 6.

### 3.1. Hemodynamics

**Figure 3:**
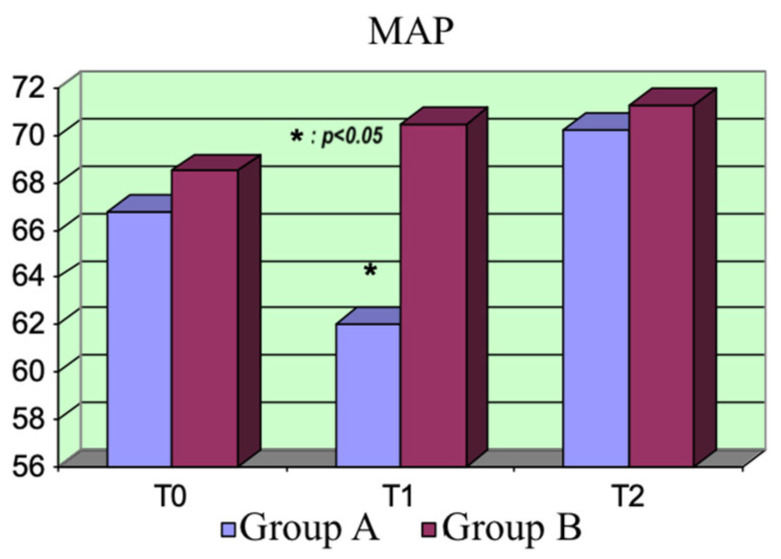
MAP variations.

Mean arterial pressure (MAP): The mean arterial pressure was not statistically different between the two groups at T_0_, but it was at T_1_ (*p* = 0.016), due to the drop in peripheral resistances after the administration of the bolus of levosimendan (in Group A, MAP T_1_ < MAP T_0_; *p* = 0.0006). Nevertheless, there was no significant difference between the two groups at T_2_. The MAP tended to moderately increase in both groups with respect to the basal values (Figure 3).

**Figure 4:**
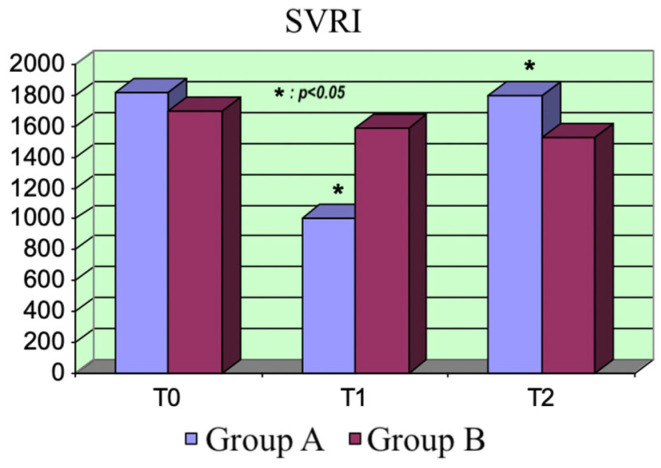
SVRI variations.

Systemic vascular resistance index (SVRI): The afterload trend reflected the MAP trend. Group A showed a transitory significative drop in the peripheral resistances after the administration of the levosimendan bolus (*p* = 0.0001). The difference between the two groups was significant at T_1_ (*p* = 0.0001). The resistances tended to grow after the bolus’s effect, exceeding those of Group B at T_2_ (*p* = 0.027). There were no differences between the SVRI values at the different treatment times in Group B (Figure 4).

**Figure 5:**
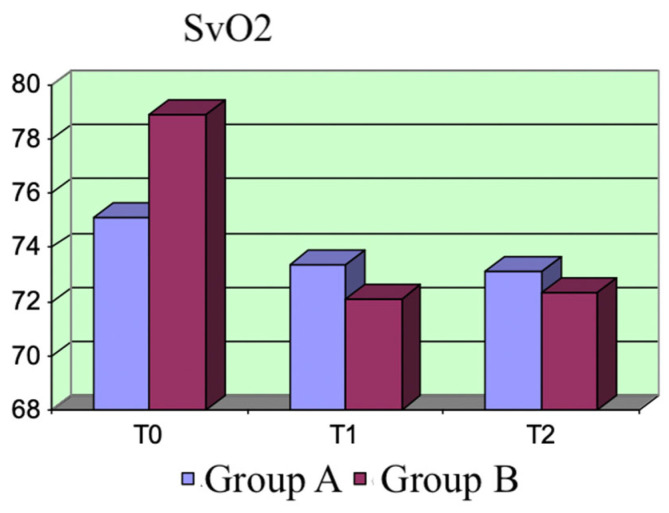
SvO_2_ variations.

Cardiac index (CI): From the beginning, there was a significant difference between the two groups (*p* = 0.008) at T_0_ because a reduced LVEF was one of the inclusion criteria for eligibility for levosimendan treatment. As expected, the CI increased in Group A patients at T_1_ (*p* = 0.0001) due to the effect of the bolus; however, it tended to reduce during the following hours, resulting in it being significantly inferior to the values for Group B at T_2_ (*p* = 0.0012). No significant variations in this parameter occurred in Group B (Figure 5).

**Figure 6:**
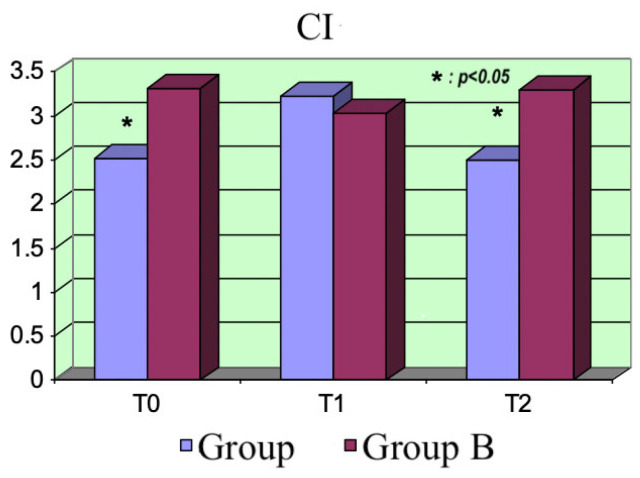
CI variations.

SvO_2_: The two groups did not significantly differ at each assessment time. We observed variations in this parameter within the same group. SvO_2_ exhibited a decreasing trend, which was more evident after the dobutamine treatment, at both T_1_ and T_2_ (T_1_: *p* = 0.01; T_2_: *p* = 0.04) (Figure 6).

**Figure 7:**
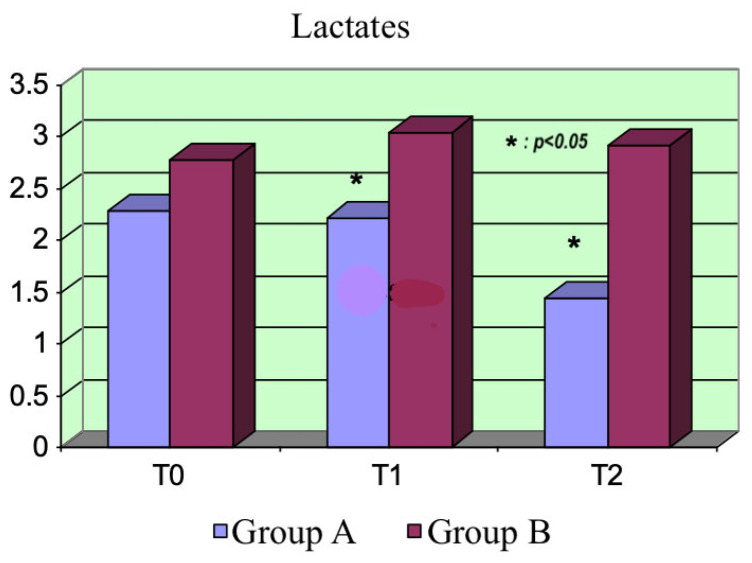
Lactate variations. Highlighted parts of the bar represent the data above mentioned in the previous paragraph.

Lactate: The hematic lactate concentration was detected with the hemogas analysis of arterial samples. Group A exhibited a decreasing trend in the hematic lactate concentration, which was evident at T_2_ (*p* = 0.0002). This result is important because a reduction in this parameter is related to better outcomes in septic patients. We did not detect this trend in Group B. The difference between the two groups was not evident at T_0_ but became significant at T_1_ and T_2_ (*p* = 0.01 and *p* = 0.0002) (Figure 7).

### 3.2. Peripheral Blood Flow

**Figure 8:**
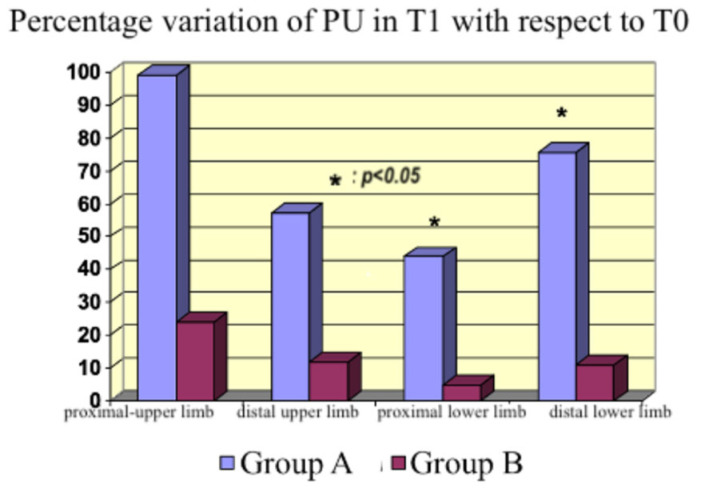
Percentage variation in PU at T_1_ with respect to T_0_.

**Figure 9:**
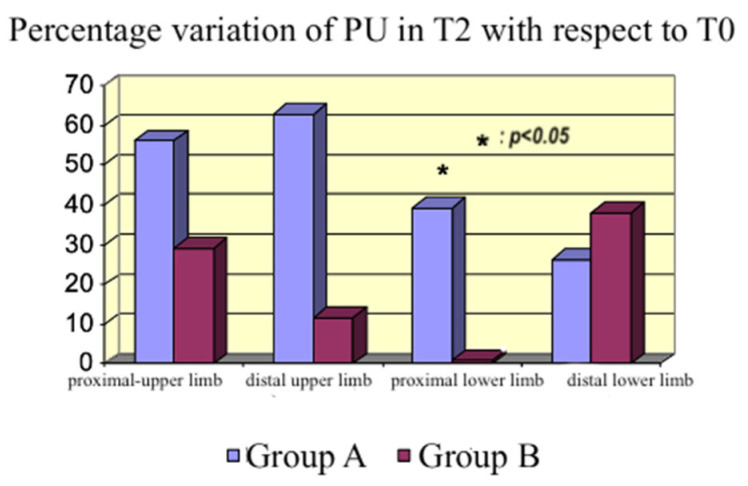
Percentage variation in PU at T_2_ with respect to T_0_.

We calculated the percentage variations in blood flow (PU) in the aforementioned areas of the body. Moreover, we compared T_1_ with T_0_ (Figure 8) and T_2_ with T_0_ (Figure 9).

An increase in the peripheral perfusion at T_1_ was observed in Group A, in both the upper and lower limbs, proximal and distal. The increase was statistically significant in the lower limb (*p* = 0.02 and *p* = 0.009). The effect persisted everywhere after 24 h of treatment, except on the distal lower limb, where we lost the significance detected at T_1_. An increase in peripheral perfusion was also observed in Group B, although it was lower than in Group A. Given that our primary endpoint was the assessment of microcirculation activity, not cardiac contractility, we could compare the two groups. The dosages of norepinephrine at T_1_ were statistically different (using the *t*-test), given the higher vasodilatory effect of the bolus of levosimendan than the continuous infusion of dobutamine. Nevertheless, microcirculation activity was not impaired by higher norepinephrine dosages, providing significant data on levosimendan’s specific action on microcirculation. 

### 3.3. Statistical Analysis

The collected data were analyzed by applying the *t*-test for non-paired data when comparing the two different groups and the *t*-test for paired data when assessing the differences within the same groups at different times. We considered statistical significance at *p* < 0.05 (Supplementary Materials). In all figures, *p* < 0.05 refers to the difference between Groups A and B.

## 4. Discussion

Microvascular dysfunction is an important element of the pathophysiology of septic shock; it may occur even in the presence of a normal systemic oxygen supply and an optimal mean arterial pressure [20]. In this context, cells are in a state of energetic failure associated with poor outcomes and organ dysfunction. Microcirculatory alterations are characterized by the presence of well-perfused areas close to non-perfused areas, leading to the distributive pattern typical of shock [8,26].

Given the reduction in hematic lactate concentrations, our findings suggest that levosimendan plays a pivotal role in restoring aerobic metabolism. Binding to the K_ATP_ channels located in mitochondria, the drug can protect mitochondria from oxidative injury, preventing calcium overload, thereby reducing mitochondrial ROS generation.

Furthermore, the drug opens adenosine triphosphate-sensitive potassium (K_ATP_) channels in vascular smooth muscle cells, inducing vasodilation. Our data showed this effect, whereby a significant increase in peripheral perfusion occurred. At higher doses, the drug also acts as a phosphodiesterase type 3 (PDE3) inhibitor. This mechanism could be responsible for the drop in peripheral resistances after administering the bolus of levosimendan at T_1_. 

The structural and functional modifications of microcirculation that occur in sepsis are considered a pivotal element in the genesis of multiorgan dysfunction. The development of a targeted therapy is needed, given the lack of treatment indications for impaired microcirculation in sepsis.

Therefore, it would be worthwhile to monitor and optimize microcirculation. In this framework, the dose, timing, and duration of therapy with inodilators should be explored and defined, as limiting microcirculation damage may reduce mortality, which remains high. 

In the context of impaired microcirculation and inadequate tissue oxygenation, inodilators improve peripheral perfusion [27,28]. An increase occurred with both drugs in our study, which was higher with levosimendan although not statistically significant at every site studied with the probe. Therefore, we cannot affirm that the flow increased in all body areas; however, we detected an increasing tendency in peripheral blood flow. The discrepancy between the measurements in the distal lower limb (Figure 8), where the dobutamine group had a higher PU% variation than the levosimendan group compared with the other sites, could be explained by the inhomogeneous endothelial damage induced by sepsis, producing a regional variability in peripheral perfusion.

The two drugs did not exhibit significant differences regarding the hemodynamic parameters. As expected, the CI increased and the peripheral resistance dropped at the beginning in Group A, consequently reducing the MAP. Moreover, the increase in peripheral blood flow was associated with reduced peripheral resistance.

The hematic lactate concentrations were significantly reduced in the levosimendan group, probably because of the enhanced aerobic metabolism due to both the action on mitochondrial K_ATP_ channels and the better oxygen delivery to cells. Contrarily, lacticemia did not change after administering dobutamine in Group B.

These results, showing a significant reduction in hematic lactate concentrations, are significant because this parameter is related to the outcomes of septic patients.

Given that the development of mitochondrial dysfunction is one of the main causes of MOF, reversing it could represent an effective therapeutic goal in sepsis treatment.

ROS production could inactivate the respiratory chain and ATP production.

Levosimendan, binding to the K_ATP_ channels in mitochondria, leads to a K^+^ flux via the mitochondrial membrane, maintaining cellular homeostasis and protecting against oxidative injury [16]. It also activates the preconditioning signaling pathways protecting against ischemia–reperfusion injury [26].

This study had limitations. It was a monocentric study including a limited number of patients. Considering an interval of confidence of 95%, a standard error of 5%, and our mean and standard deviation results, the ideal sample size could be 45 patients. The two groups differed because we included patients with a previously documented reduction in the ejection fraction of the left ventricle in the levosimendan group; conversely, we preserved the previous left ventricle ejection fraction of patients in the dobutamine group. However, this study’s primary endpoint was the assessment of the two drugs on peripheral perfusion using the PU, not their inotropic effect, and we compared their effects concerning only the dilator effect on peripheral perfusion.

## 5. Conclusions

In conclusion, our results agree with the data from the literature. There is no evidence of levosimendan’s superiority over dobutamine in patients with sepsis and septic shock [21,22]. Nonetheless, employing an inodilator could restore the microcirculation function and improve tissue oxygenation. The difference we detected between the two drugs was that levosimendan led to a higher increase in peripheral blood flow and a significant reduction in the hematic lactate concentration, which indicates higher aerobic metabolic activity. Hence, levosimendan could be indicated in septic patients with impaired microcirculation and tissue oxygenation, and high lactate levels. This may be an interesting perspective for the future of intensive care in sepsis management. Nevertheless, this was a pilot study, and more data and larger studies are needed before more definitive conclusions can be drawn.

## Figures and Tables

**Table 1:** Patients belonging to group A.

Patient	Age (yrs)	Sex	Comorbidities	Cause of Sepsis	SAPS II	APACHE II	Mechanical Ventilation	Dose of Norepinephrine at T_0_ (mcg/kg/min)	Dose of Norepinephrine at T_1_ (mcg/kg/min)	Dose of Norepinephrine at T_2_ (mcg/kg/min)
1	78	M	Metabolic syndrome; Gilbert syndrome	Cholecystitis	41	25	YES	0.25	0.4	0.3
2	64	F	Rheumatoid arthritis	Post-traumatic fascitis of the right forearm associated with bone fractures	45	21	YES	0.3	0.35	0.25
3	42	F	Insufficient aortic valve; anemia	Nephrolithiasis with pyelonephritis	25	9	YES	0.25	0.4	0.2
4	77	M	Arterial hypertension; previous myocardial infarction	Bowel perforation	32	19	YES	0.15	0.25	0.2
5	53	F	Obesity (BMI = 40); smoker	Urinary tract infection	35	15	YES	0.3	0.4	0.2
6	81	M	Benign prostatic hyperplasia; remote coronary angioplasty	Wound dehiscence after right hemicolectomy	74	25	YES	0.25	0.4	0.3
7	74	M	Obesity (BMI = 35); hypertension; and glucose intolerance	Cholangitis	81	26	YES	0.35	0.4	0.4
8	48	M	Smoker; mild diabetes mellitus 2; and hypertensive cardiomyopathy	Diverticulitis with bowel perforation	53	17	YES	0.1	0.25	0.2
Median value	65.5				43	20		0.25	0.4	0.225

SAPS II: Simplified Acute Physiology Score; APACHE II: Acute Physiology and Chronic Health Evaluation.

**Table 2:** Patients belonging to group B.

Patient	Age (yrs)	Sex	Comorbidities	Cause of Sepsis	SAPS II	APACHE II	Mechanical Ventilation	Dose of Norepinephrine at T_0_ (mcg/kg/min)	Dose of Norepinephrine at T_1_ (mcg/kg/min)	Dose of Norepinephrine at T_2_ (mcg/kg/min)
9	71	M	Arterial hypertension	Stercoral peritonitis	47	24	YES	0.1	0.2	0.2
10	58	F	None	Nephrolithiasis	25	14	YES	0.2	0.2	0.2
11	65	M	Glucose intolerance	Iatrogenic bowel perforation	35	23	YES	0.15	0.3	0.2
12	55	M	Smoker; arterial hypertension	Cholangitis	38	19	YES	0.1	0.15	0.05
13	74	M	Chronic lymphatic leukemia	Fascitis of the neck from a tooth abscess	27	11	YES	0.5	0.1	0.1
14	83	M	Arterial hypertension; admission to the hospital for hemorrhagic stroke	Device-related infection	41	26	YES	0.2	0.4	0.25
15	82	F	Arterial hypertension; previous deep vein thrombosis	Erysipelas on the left lower limb	26	10	YES	0.1	0.25	0.15
16	73	M	Arterial hypertension; initial cognitive impairment	Nephrolithiasis with pyelonephritis	74	21	YES	0.3	0.4	0.4
Median value	73				35	19		0.2	0.25	0.2

SAPS II: Simplified Acute Physiology Score; APACHE II: Acute Physiology and Chronic Health Evaluation.

**Table 3:** Hemodynamic parameters in group A. Abbreviations: MAP: mean arterial pressure; HR: heart rate; SaO_2_: arterial saturation of oxygen; SvO_2_: venous saturation of oxygen; CI: Cardiac Index; SVRI: systemic vascular resistance index; ELWI: extravascular lung water index; ITBVI: intrathoracic blood volume index; GEDVI: Global End-Diastolic Volume Index; σ: standard deviation.

	T_0_		T_1_		T_2_	
	Mean	σ	Mean	σ	Mean	σ
MAP (mmHg)	66.8	2.95	62	4.07	70.25	9.36
HR (bpm)	104	13.47	104.2	11.53	97.25	13.03
SaO_2_ (%)	97.4	0.41	97.8	0.99	98.5	1.19
SvO_2_ (%)	75.12	11.62	73.38	9.87	73.12	6.73
CI	2.52	0.37	3.22	0.44	2.5	0.49
SVRI	1821.2	335.71	1001.8	202.79	1795.12	248.28
ELWI	12.76	1.02	12.1	1.57	10.41	1.35
ITBVI	1094.6	52.96	1088.2	27.61	1049.12	87.53
GEDVI	869.48	42.06	863.97	21.9	830.28	69.27
Lactate (mmol/L)	2.28	0.25	2.21	0.24	1.45	0.31

**Table 4:** Peripheral blood flow (PU) variations in group A. σ: standard deviation.

Blood Flow (PU)	Proximal Upper Limb		Distal Lower Limb		Proximal Upper Limb		Distal Lower Limb	
	Mean	σ	Mean	σ	Mean	σ	Mean	σ
T_0_	11.05	2.84	11.97	6.11	11.05	2.84	11.97	6.11
T_1_	20.6	8.04	17.47	7.31	20.6	8.04	17.47	7.31
T_2_	16.28	4.27	17.03	7.55	16.28	4.27	17.03	7.55

**Table 5:** Hemodynamic parameters in group B. Abbreviations: MAP: mean arterial pressure; HR: heart rate; SaO_2_: arterial saturation of oxygen; SvO_2_: venous saturation of oxygen; CI: Cardiac Index; SVRI: systemic vascular resistance index; ELWI: extravascular lung water index; ITBVI: intrathoracic blood volume index; GEDVI: Global End-Diastolic Volume Index; σ: standard deviation.

	T_0_		T_1_		T_2_	
	Mean	σ	Mean	σ	Mean	σ
MAP (mmHg)	68.5	15.93	70.5	7.87	71.25	6.04
HR (bpm)	120.37	9.07	116.62	5.37	114.5	7.79
SaO_2_ (%)	98	1.2	98	0.75	98.25	1.16
SvO_2_ (%)	78.92	4.92	72.12	5.62	72.37	5.83
CI	3.33	0.65	3.03	0.28	3.3	0.25
SVRI	1701.37	261.4	1586.62	147.78	1529.37	177.44
ELWI	7.2	2	6.9	1.24	7.27	1.02
ITBVI	961.25	388.59	1075.12	167.48	1060.5	162.42
GEDVI	754.53	305.02	852.69	131.84	840.09	128.31
Lactate (mmol/L)	2.79	0.91	3.04	0.74	2.92	0.76

**Table 6:** Peripheral blood flow (PU) variations in group B. σ: standard deviation.

Blood Flow (PU)	Proximal Upper Limb		Distal Upper Limb		Proximal Lower Limb		Distal Lower Limb	
	Mean	σ	Mean	σ	Mean	σ	Mean	σ
T_0_	12.52	5.66	11.65	3.7	10.37	3.44	8.58	3.46
T_1_	14.78	6.39	13.35	5.28	8.81	2.4	8.08	3.05
T_2_	16.69	7.87	14.19	6.35	11.88	7.03	9.38	3.81

## Data Availability

All data generated or analyzed during this study are included in this published article. The data supporting the findings of this study are openly available.

## Supplementary Materials

The following supporting information can be downloaded at https://www.mdpi.com/article/10.3390/life15060871/s1. Supplementary Material S1: Statistical analysis.

## Abbreviations

MAP	mean arterial pressure
HR	heart rate
SaO_2_	arterial saturation of oxygen
SvO_2_	venous saturation of oxygen
CI	cardiac index
SVRI	systemic vascular resistance index
ELWI	extravascular lung water index
ITBVI	intrathoracic blood volume index
σ	standard deviation
