# Supplementary material for: The Effects of Levosimendan on Microcirculation and Peripheral Perfusion in Septic Shock: A Pilot Study
Source: Life (Basel). 2025 May 28;15(6):871. doi: 10.3390/life15060871 (PMC12194462; doi:10.3390/life15060871)
Supplement: Supplementary file 1 [file life-15-00871-s001.zip › life-3126982-supplementary.pdf]

Peripheral blood flow data

Proximal upper limb

```
Sample 1
Variable    : T0

Sample size                  =       8
Lowest value                 =         7,2000
Highest value                =        15,3000
Arithmetic mean              =        11,0460
  95% CI for the mean        =         9,0132 to 13,0788
Standard deviation           =         2,8417
Standard error of the mean   =         0,8986
------------------------------------------------------------
Sample 2
Variable    : T1

Sample size                  =        8
Lowest value                 =         8,9000
Highest value                =        35,0000
Arithmetic mean              =        20,5970
  95% CI for the mean        =        14,8481 to 26,3459
Standard deviation           =         8,0364
Standard error of the mean   =         2,5413
------------------------------------------------------------
Paired t-test

Mean difference    : -9,5510
Standard deviation : 8,5917
95 % CI            : -15,6971 to -3,4049
t=-3,515  DF=9  P = 0,0066

Sample 1
Variable    : T0

Sample size                  =        8
Lowest value                 =         7,2000
Highest value                =        15,3000
Arithmetic mean              =        11,0460
  95% CI for the mean        =         9,0132 to 13,0788
Standard deviation           =         2,8417
Standard error of the mean   =         0,8986
------------------------------------------------------------
Sample 2
Variable    : T2

Sample size                  =        8
Lowest value                 =         7,4000
Highest value                =        23,1000
Arithmetic mean              =        16,2850
  95% CI for the mean        =        13,2321 to 19,3379
Standard deviation           =         4,2677
Standard error of the mean   =         1,3496
------------------------------------------------------------
Paired t-test

Mean difference    : -5,2390
Standard deviation : 4,8216
95 % CI            : -8,6881 to -1,7899
t=-3,436  DF=9  P = 0,0074
```

Distal upper limb

```
Sample 1

Variable    : T0
Sample size                  =        8
Lowest value                 =         5,4000
Highest value                =        27,3000
Arithmetic mean              =        11,9760
  95% CI for the mean        =         7,6015 to 16,3505
Standard deviation           =         6,1151
Standard error of the mean   =         1,9338
------------------------------------------------------------
Sample 2
Variable    : T1

Sample size                  =        8
Lowest value                 =        13,0000
Highest value                =        23,0000
Arithmetic mean              =        17,4500
  95% CI for the mean        =        14,8904 to 20,0096
Standard deviation           =         3,5781
Standard error of the mean   =         1,1315
------------------------------------------------------------
Paired t-test

Mean difference    : -5,4740
Standard deviation : 7,1307
95 % CI            : -10,5750 to -0,3730
t=-2,428  DF=9  P = 0,0381
```

```
Sample 1
Variable    : T0

Sample size                  =        8
Lowest value                 =         5,4000
Highest value                =        27,3000
Arithmetic mean              =        11,9760
  95% CI for the mean        =         7,6015 to 16,3505
Standard deviation           =         6,1151
Standard error of the mean   =         1,9338
------------------------------------------------------------
Sample 2
Variable    : T2

Sample size                  =        8
Lowest value                 =        12,0000
Highest value                =        22,0000
Arithmetic mean              =        17,0400
  95% CI for the mean        =        14,3561 to 19,7239
Standard deviation           =         3,7518
Standard error of the mean   =         1,1864
------------------------------------------------------------
Paired t-test

Mean difference    : -5,0640
Standard deviation : 6,5182
95 % CI            : -9,7268 to -0,4012
t=-2,457  DF=9  P = 0,0363
```

Proximal lower limb

```
Sample 1
Variable    : T0

Sample size                  =        8
Lowest value                 =         5,9000
Highest value                =        16,3000
Arithmetic mean              =        10,2340
  95% CI for the mean        =         7,4948 to 12,9732
Standard deviation           =         3,8291
Standard error of the mean   =         1,2109
------------------------------------------------------------
Sample 2
Variable    : T1

Sample size                  =        8
Lowest value                 =        11,0000
Highest value                =        21,0000
Arithmetic mean              =        14,3000
  95% CI for the mean        =        11,5438 to 17,0562
Standard deviation           =         3,8528
Standard error of the mean   =         1,2184
------------------------------------------------------------
Paired t-test

Mean difference    : -4,0660
Standard deviation : 3,6257
95 % CI            : -6,6597 to -1,4723
t=-3,546  DF=9  P = 0,0063

Sample 1
Variable    : T0

Sample size                  =        8
Lowest value                 =         5,9000
Highest value                =        16,3000
Arithmetic mean              =        10,2340
  95% CI for the mean        =         7,4948 to 12,9732
Standard deviation           =         3,8291
Standard error of the mean   =         1,2109
------------------------------------------------------------
Sample 2
Variable    : T2

Sample size                  =        8
Lowest value                 =         7,4000
Highest value                =        20,0000
Arithmetic mean              =        14,0400
  95% CI for the mean        =        11,1767 to 16,9033
Standard deviation           =         4,0027
Standard error of the mean   =         1,2658
------------------------------------------------------------
Paired t-test

Mean difference    : -3,8060
Standard deviation : 1,4954
95 % CI            : -4,8757 to -2,7363
t=-8,048  DF=9  P < 0,0001
```

DISTAL lower limb

```
Variable    : T0

Sample size                   =        8
Lowest value                  =         4,4000
Highest value                 =         9,9000
Arithmetic mean               =         7,3170
  95% CI for the mean         =         5,9288 to 8,7052
Standard deviation            =         1,9406
Standard error of the mean    =         0,6137
------------------------------------------------------------
Sample 2
Variable    : T1

Sample size                   =        8
Lowest value                  =         9,4000
Highest value                 =        17,5000
Arithmetic mean               =        12,4670
  95% CI for the mean         =        10,3912 to 14,5428
Standard deviation            =         2,9018
Standard error of the mean    =         0,9176
------------------------------------------------------------
Paired t-test

Mean difference    : -5,1500
Standard deviation : 2,6996
95 % CI            : -7,0812 to -3,2188
t=-6,033  DF=9  P = 0,0002
```

```
Sample 1
Variable    : T0

Sample size                   =        8
Lowest value                  =         4,4000
Highest value                 =         9,9000
Arithmetic mean               =         7,3170
  95% CI for the mean         =         5,9288 to 8,7052
Standard deviation            =         1,9406
Standard error of the mean    =         0,6137
------------------------------------------------------------
Sample 2
Variable    : T2

Sample size                   =        8
Lowest value                  =         6,0000
Highest value                 =        13,0000
Arithmetic mean               =         9,1900
  95% CI for the mean         =         7,4674 to 10,9126
Standard deviation            =         2,4081
Standard error of the mean    =         0,7615
------------------------------------------------------------
Paired t-test

Mean difference    : -1,8730
Standard deviation : 1,6979
95 % CI            : -3,0876 to -0,6584
t=-3,488  DF=9  P = 0,0068
```

Sample 1

Hemodynamic variables

```
Sample 1
Variable    : CI

Sample size                  =         8
Lowest value                 =         1.8400
Highest value                =         3.2300
Arithmetic mean              =         2.5200
  95% CI for the mean        =         2.2092 to 2.8308
Standard deviation           =         0.3718
Standard error of the mean   =         0.1314
------------------------------------------------------------
Sample 2
Variable    : Cib

Sample size                  =         8
Lowest value                 =         2.2000
Highest value                =         3.2000
Arithmetic mean              =         2.6125
  95% CI for the mean        =         2.3580 to 2.8670
Standard deviation           =         0.3044
Standard error of the mean   =         0.1076
------------------------------------------------------------
Paired t-test

Mean difference    : -0.0925
Standard deviation : 0.1821
95 % CI            : -0.2447 to 0.0597
t=-1.437  DF=7  P = 0.1940
```

```
Sample 1
Variable    : CI

Sample size                  =         8
Lowest value                 =         1.8400
Highest value                =         3.2300
Arithmetic mean              =         2.5200
  95% CI for the mean        =         2.2092 to 2.8308
Standard deviation           =         0.3718
Standard error of the mean   =         0.1314
------------------------------------------------------------
Sample 2
Variable    : Cif

Sample size                  =         8
Lowest value                 =         2.9000
Highest value                =         3.8000
Arithmetic mean              =         3.2500
  95% CI for the mean        =         3.0135 to 3.4865
Standard deviation           =         0.2828
Standard error of the mean   =         0.1000
------------------------------------------------------------
Paired t-test

Mean difference    : -0.7300
Standard deviation : 0.5798
95 % CI            : -1.2147 to -0.2453
t=-3.561  DF=7  P = 0.0092
```

```
Sample 1
Variable    : CI

Sample size                   =         8
Lowest value                  =         1.8400
Highest value                 =         3.2300
Arithmetic mean               =         2.5200
  95% CI for the mean         =         2.2092 to 2.8308
Standard deviation            =         0.3718
Standard error of the mean    =         0.1314
------------------------------------------------------------
Sample 2
Variable    : Cifinf

Sample size                   =         8
Lowest value                  =         2.9000
Highest value                 =         3.8000
Arithmetic mean               =         3.2500
  95% CI for the mean         =         3.0135 to 3.4865
Standard deviation            =         0.2828
Standard error of the mean    =         0.1000
------------------------------------------------------------
Paired t-test

Mean difference    : -0.7300
Standard deviation : 0.5798
95 % CI            : -1.2147 to -0.2453
t=-3.561  DF=7  P = 0.0092



Sample 1
Variable    : PAM

Sample size                   =         8
Lowest value                  =        60.0000
Highest value                 =        70.0000
Arithmetic mean               =        66.8000
  95% CI for the mean         =        64.3361 to 69.2639
Standard deviation            =         2.9472
Standard error of the mean    =         1.0420
------------------------------------------------------------
Sample 2
Variable    : pamb

Sample size                   =         8
Lowest value                  =        54.0000
Highest value                 =        67.0000
Arithmetic mean               =        62.0000
  95% CI for the mean         =        58.5967 to 65.4033
Standard deviation            =         4.0708
Standard error of the mean    =         1.4392
------------------------------------------------------------
Paired t-test

Mean difference    : 4.8000
Standard deviation : 2.2928
95 % CI            : 2.8831 to 6.7169
t=5.921  DF=7  P = 0.0006
```

```
Sample 1
Variable    : PAM

Sample size                   =         8
Lowest value                  =       60.0000
Highest value                 =       70.0000
Arithmetic mean               =       66.8000
  95% CI for the mean         =       64.3361 to 69.2639
Standard deviation            =        2.9472
Standard error of the mean    =        1.0420
------------------------------------------------------------
Sample 2
Variable    : pamf

Sample size                   =         8
Lowest value                  =       61.0000
Highest value                 =       90.0000
Arithmetic mean               =       70.2500
  95% CI for the mean         =       62.4234 to 78.0766
Standard deviation            =        9.3618
Standard error of the mean    =        3.3099
------------------------------------------------------------
Paired t-test

Mean difference    : -3.4500
Standard deviation : 8.8277
95 % CI            : -10.8302 to 3.9302
t=-1.105  DF=7  P = 0.3055


Sample 1
Variable    : PAM

Sample size                   =         8
Lowest value                  =       60.0000
Highest value                 =       70.0000
Arithmetic mean               =       66.8000
  95% CI for the mean         =       64.3361 to 69.2639
Standard deviation            =        2.9472
Standard error of the mean    =        1.0420
------------------------------------------------------------
Sample 2
Variable    : pamfininf

Sample size                   =         8
Lowest value                  =       64.0000
Highest value                 =       81.0000
Arithmetic mean               =       71.0000
  95% CI for the mean         =       66.4428 to 75.5572
Standard deviation            =        5.4511
Standard error of the mean    =        1.9272
------------------------------------------------------------
Paired t-test

Mean difference    : -4.2000
Standard deviation : 5.9714
95 % CI            : -9.1922 to 0.7922
t=-1.989  DF=7  P = 0.0870
```

```
Sample 1
Variable    : SvO2

Sample size                   =         8
Lowest value                  =       58.0000
Highest value                 =       90.0000
Arithmetic mean               =       76.6250
  95% CI for the mean         =       66.9480 to 86.3020
Standard deviation            =       11.5751
Standard error of the mean    =        4.0924
------------------------------------------------------------
Sample 2
Variable    : svo2b

Sample size                   =         8
Lowest value                  =       52.0000
Highest value                 =       88.0000
Arithmetic mean               =       73.1250
  95% CI for the mean         =       64.8195 to 81.4305
Standard deviation            =        9.9346
Standard error of the mean    =        3.5124
------------------------------------------------------------
Paired t-test

Mean difference    : 3.5000
Standard deviation : 8.9762
95 % CI            : -4.0043 to 11.0043
t=1.103  DF=7  P = 0.3066
```

```
Sample 1
Variable    : SvO2

Sample size                   =         8
Lowest value                  =       58.0000
Highest value                 =       90.0000
Arithmetic mean               =       76.6250
  95% CI for the mean         =       66.9480 to 86.3020
Standard deviation            =       11.5751
Standard error of the mean    =        4.0924
------------------------------------------------------------
Sample 2
Variable    : svo2f

Sample size                   =         8
Lowest value                  =       65.0000
Highest value                 =       79.0000
Arithmetic mean               =       73.0000
  95% CI for the mean         =       68.9534 to 77.0466
Standard deviation            =        4.8403
Standard error of the mean    =        1.7113
------------------------------------------------------------
Paired t-test

Mean difference    : 3.6250
Standard deviation : 10.3501
95 % CI            : -5.0279 to 12.2779
t=0.991  DF=7  P = 0.3549
```

```
Sample 1
Variable    : SvO2

Sample size                   =         8
Lowest value                  =       58.0000
Highest value                 =       90.0000
Arithmetic mean               =       76.6250
  95% CI for the mean         =       66.9480 to 86.3020
Standard deviation            =       11.5751
Standard error of the mean    =        4.0924
------------------------------------------------------------
Sample 2
Variable    : svo2finf

Sample size                   =         8
Lowest value                  =       67.0000
Highest value                 =       76.0000
Arithmetic mean               =       72.8750
  95% CI for the mean         =       70.0687 to 75.6813
Standard deviation            =        3.3568
Standard error of the mean    =        1.1868
------------------------------------------------------------
Paired t-test

Mean difference    : 3.7500
Standard deviation : 11.3358
95 % CI            : -5.7270 to 13.2270
t=0.936  DF=7  P = 0.3806



Sample 1
Variable    : Lattati

Sample size                   =         8
Lowest value                  =        1.8000
Highest value                 =        3.1000
Arithmetic mean               =        2.4188
  95% CI for the mean         =        2.1048 to 2.7327
Standard deviation            =        0.3756
Standard error of the mean    =        0.1328
------------------------------------------------------------
Sample 2
Variable    : latb

Sample size                   =         8
Lowest value                  =        1.8000
Highest value                 =        2.7000
Arithmetic mean               =        2.2160
  95% CI for the mean         =        2.0128 to 2.4192
Standard deviation            =        0.2431
Standard error of the mean    =        0.0860
------------------------------------------------------------
Paired t-test

Mean difference    : 0.2028
Standard deviation : 0.3911
95 % CI            : -0.1242 to 0.5297
t=1.466  DF=7  P = 0.1860
```

```
Sample 1
Variable    : Lattati

Sample size                   =        8
Lowest value                  =        1.8000
Highest value                 =        3.1000
Arithmetic mean               =        2.4188
  95% CI for the mean         =        2.1048 to 2.7327
Standard deviation            =        0.3756
Standard error of the mean    =        0.1328
------------------------------------------------------------
Sample 2
Variable    : latf

Sample size                   =        8
Lowest value                  =        1.1200
Highest value                 =        2.0100
Arithmetic mean               =        1.4525
  95% CI for the mean         =        1.1965 to 1.7085
Standard deviation            =        0.3063
Standard error of the mean    =        0.1083
------------------------------------------------------------
Paired t-test

Mean difference    : 0.9663
Standard deviation : 0.4243
95 % CI            : 0.6115 to 1.3210
t=6.441  DF=7  P = 0.0004


Sample 1
Variable    : Lattati

Sample size                   =        8
Lowest value                  =        1.8000
Highest value                 =        3.1000
Arithmetic mean               =        2.4188
  95% CI for the mean         =        2.1048 to 2.7327
Standard deviation            =        0.3756
Standard error of the mean    =        0.1328
------------------------------------------------------------
Sample 2
Variable    : latfinf

Sample size                   =        8
Lowest value                  =        0.8900
Highest value                 =        1.4000
Arithmetic mean               =        1.1713
  95% CI for the mean         =        1.0272 to 1.3153
Standard deviation            =        0.1723
Standard error of the mean    =        0.0609
------------------------------------------------------------
Paired t-test

Mean difference    : 1.2475
Standard deviation : 0.3892
95 % CI            : 0.9221 to 1.5729
t=9.065  DF=7  P < 0.0001
```
